# Endothelium-Dependent Vasorelaxant Effect of Butanolic Fraction from *Caryocar brasiliense* Camb. Leaves in Rat Thoracic Aorta

**DOI:** 10.1155/2012/934142

**Published:** 2012-08-15

**Authors:** Lais Moraes de Oliveira, Aline Gabriela Rodrigues, Elaine Fernanda da Silva, Letícia Bonancio Cerqueira, Carlos Henrique Castro, Gustavo Rodrigues Pedrino, Maria Helena Catelli de Carvalho, Roberto Pontarolo, Elson Alves Costa, Francinete Ramos Campos, Fernando Paranaiba Filgueira, Paulo César Ghedini

**Affiliations:** ^1^Department of Physiological Sciences, Laboratory of Molecular and Biochemistry Pharmacology, Institute of Biological Sciences, Federal University of Goiás, Campus Samambaia, Sala 215, 74001-970 Goiânia, GO, Brazil; ^2^Department of Physiological Sciences, Laboratory of Autonomic and Cardiac Physiology, Federal University of Goiás, 74001-970 Goiânia, GO, Brazil; ^3^Department of Pharmacy, Study Center in Biopharmacy, Federal University of Paraná, 80210-170 Curitiba, PR, Brazil; ^4^Department of Physiological Sciences, Laboratory of Physiology of Isolated Organs, Federal University of Goiás, 74001-970 Goiânia, GO, Brazil; ^5^Department of Pharmacology, Laboratory of Hypertension, University of São Paulo, 05508-900 São Paulo, SP, Brazil; ^6^Department of Physiological Sciences, Laboratory of Pharmacology of Natural Products, Federal University of Goiás, 74001-970 Goiânia, GO, Brazil

## Abstract

*Caryocar brasiliense* Camb. “pequi” is a native plant from the Cerrado region of Brazil that contains bioactive components reported to be antioxidant agents. Previous work has demonstrated that dietary supplementation with pequi decreased the arterial pressure of volunteer athletes. We found that the crude hydroalcoholic extract (CHE) of *C. brasiliense* leaves relaxed, in a concentration-dependent manner, rat aortic rings precontracted with phenylephrine, and that the butanolic fraction (BF) produced an effect similar to that of the CHE. Aortic relaxation induced by BF was abolished by endothelium removal, by incubation of the nitric oxide synthase inhibitor L-NAME, or the soluble guanylatecyclase inhibitor ODQ. However, incubation with atropine and pyrilamine had no effect on the BF-induced vasorelaxation. Moreover, this effect was not inhibited by indomethacin and tetraethylammonium. The concentration-response curve to calcium in denuded-endothelium rings was not modified after incubation with BF, and the vasorelaxation by BF in endothelium-intact rings precontracted with KCl was abolished after incubation with L-NAME. In addition, administration of BF in anesthetized rats resulted in a reversible hypotension. The results reveal that *C. brasiliense* possesses both in vivo and in vitro activities and that the vascular effect of BF involves stimulation of the nitric oxide/cyclic GMP pathway.

## 1. Introduction

*Caryocar brasiliense *Camb., known as “pequi,” is a tree that belongs to the Caryocaraceae family and is widely distributed in the Cerrado region of Brazil [1, 2]. Pequi fruit are rich in A and E vitamins [3] and are popularly used to flavor cuisine. Oil extracted from the fruits is used in the cosmetic industry for the production of soaps and skin creams and is a useful tool in the production of fuel and lubricants [4, 5]. In folk medicine, pequi fruit is used against colds and flu. The seed fat is used for the treatment of respiratory tract diseases, such as asthma and bronchitis, whereas a decoction of the leaves and flowers is used to boost energy, as an aphrodisiac, and for the treatment of liver disease. The bark and fruit are used as febrifuges and diuretics [3]. 

Studies exploring the biological effects of *C. brasiliense* have shown that the hydroethanolic extract of pequi leaves presents leishmanicidal, antibacterial, and antioxidant effects [6]. In addition, the aqueous extract of pequi fruit pulp has revealed anticlastogenic potential, antigenotoxic activity, and antioxidant properties [7, 8].

The chemical characterization of polar components of pequi ethanolic extracts revealed the presence of bioactive components widely reported as antioxidant agents, including gallic acid, quinic acid, quercetin, and quercetin 3-*O*-arabinose [9]. Several studies suggest that increased intake of antioxidant compounds may help to protect against chronic pathologies, such as cardiovascular diseases [10–14], and a recent study has shown that dietary supplementation with pequi decreased the arterial pressure of volunteer athletes when compared to a control group [15].

The aim of the present study was to evaluate the vasorelaxant effect of the crude hydroalcoholic extract (CHE) from *C. brasiliense* leaves in rat thoracic aorta, to determine the active organic fractions of CHE, and to investigate the mechanisms of action. In addition, we analyzed the in vivo effect of the extract of the active organic fraction.

## 2. Material and Methods

### 2.1. Chemicals, Reagents, and Standards

Phenylephrine (Phe), acetylcholine (ACh), sodium nitroprusside (SNP), N*ω*-nitro-L-arginine methyl ester (L-NAME), tetraethylammonium (TEA), indomethacin, 1H-[1,2, 4]oxadiazolo[4,3-alpha]quinoxalin-1-one (ODQ), atropine, pyrilamine, standards of gallic acid (97.5–102.5%), and quercetin (≥98%) were purchased from Sigma-Aldrich Co. (St. Louis, MO, USA). Methanol, acetonitrile (HPLC grade), and formic acid (88%) were purchased from J. T. Baker Chemicals B. V. (Deventer, The Netherlands), and ammonium formate was purchased from Acros Organics (New Jersey, USA). Ultrapure water was obtained using a Milli-Q purification system from Millipore (Bedford, MA, USA).

### 2.2. Plant Material

Leaves of *C. brasiliense *Camb. were collected on September 2010 in Gurupi, Tocantins, Brazil. The plant was authenticated by Proffessor Aristônio Magalhães Teles, and a voucher specimen was deposited at the herbarium of the Institute of Biological Sciences of the Federal University of Goiás under number 1353. The leaves were maintained at room temperature (35°C) for drying and stabilization. The plant material was pulverized in a Willye mill.

### 2.3. Crude Hydroalcoholic Extract Preparation

To prepare the crude hydroalcoholic extract (CHE) of *C. brasiliense* leaves, 50 g of powdered leaves were immersed in 1000 mL of an ethanol-water solution (70 : 30, v/v), for 48–72 h, with occasional stirring. The extract was then vacuum-filtered, concentrated in a rotary evaporator at reduced pressure and at a temperature lower than 50°C, lyophilized and stored in the dark at 4°C.

### 2.4. Crude Hydroalcoholic Extract Fractionation

The CHE (10 g) was dissolved in water (500 mL) and extracted with n-hexane (3 × 300 mL) at room temperature. After the removal of the hexane fraction, the remaining aqueous extract was eluated with chloroform (3 × 300 mL). After the removal of the chloroform fraction, the remaining aqueous extract was eluated with ethyl acetate (3 × 300 mL). After removal of the ethyl acetate fraction, the remaining aqueous layer was extracted with n-butanol (3 × 300 mL). Then, after the removal of the n-butanol fraction, each of the fractions (except the final aqueous portion) was separately evaporated to dryness under reduced pressure to give a hexane fraction (HF) (0.29 g), a chloroform fraction (CF) (0.37 g), an ethyl acetate fraction (EAF) (1.76 g), and an n-butanol fraction (BF) (1.37 g). The final aqueous layer was disposed of.

### 2.5. Standards and BF Preparation for LC-MS Analysis

Stock solutions of gallic acid and quercetin standards were prepared separately in methanol at concentrations of 1 mg/mL. All stock solutions were stored under refrigeration at 4°C. Working solutions were obtained from stock solutions by appropriate dilution in methanol/water solution (70 : 30 v/v) containing 1% formic acid or 1 mM ammonium formate, depended of each experiment, to the final concentration of 0.5 *μ*g/mL, 50 *μ*g/mL and 2 mg/mL of gallic acid, and quercetin and BF solutions, respectively. All working standard solutions and samples were filtered through a polyvinylidene fluoride (PVDF) syringe filter (11 mm, 0.45 mm, Millipore Millex, Billerica, MA, USA) before injection into the LC-MS system.

### 2.6. LC-MS Instrumentation and Conditions

LC-ESIMS analyses were performed using an Agilent 1200 RR-HT System (Wilmington, NC, USA) that consisted of a G1311A binary pump, G1379A degasser, and G1316A column oven. These were connected with a CTC Sample Manager (Model 2777, Waters, Milford, CT, USA). The system was coupled to an Applied Biosystems MDS Sciex API 3200 Triple Quadrupole Mass Spectrometer (Toronto, Canada) equipped with a syringe pump Harvard 22 Dual Model (Harvard Apparatus, South Natick, MA, USA) and an Electrospray ionization (ESI) source. The ESI source was operated in the positive and negative ion mode for monitoring quercetin and in the negative ion mode. For monitoring gallic acid in the butanolic fraction. For the positive ion mode, the mobile phase consisted of methanol/water solution (10 : 90 v/v) (A) and acetonitrile (B), both containing 1% formic acid. The flow rate was maintained at 300 *μ*L/min, and the gradient profile was as follows: *t*_0–5.0 min⁡_: A = 100% to 90%; *t*_5.0–35.0 min⁡_: A = 90% to 30%; *t*_35.0–40.0 min⁡_: A = 30%; *t*_40.0–41.0 min⁡_: A = 30% to 100%; *t*_41.0–46.0 min⁡_: A = 100%. For the negative ion mode, the mobile phase consisted of methanol/water solution (10 : 90 v/v) (A) and acetonitrile (B) both containing 1 mM ammonium formate. The flow rate and the gradient profile were the same used for the positive ion mode. For both methods, the analyte separations were achieved on an XBridge C18 150 × 2.1 mm (5 mm particle size) column coupled with an XBridge C18 10 × 2.1 mm (5 mm particle size) guard column. The injection volume was 20 *μ*L, and the column temperature was maintained at 25°C. Data acquisition was performed with the MS Workstation by Analyst 1.4 software (ABI/Sciex). The MS and ion-source parameters for ESI-positive mode were the following: declustering potential (DP), 40; entrance potential (EP), 10; curtain gas (CUR), 10 psi; ion spray voltage (IS), 5500 V; nebulizer gas (GS1), 45 psi; turbo gas (GS2), 45 psi; temperature, 400°C. The MS and ion source parameters for ESI negative mode were as follows: declustering potential (DP), −40; entrance potential (EP), −10; CUR, 10 psi; IS, −4500 V; GS1, 45 psi; GS2, 45 psi; temperature, 400°C. The high-purity nitrogen and zero grade air that were used as the CUR, GS1 and GS2 gases were produced by a high-purity nitrogen generator from PEAK Scientific Instruments (Chicago, IL, USA).

### 2.7. Animals

Male Wistar rats (200–300 g) from the colony of the Federal University of Goiás were used in this study. The animals were housed under a controlled 12 h light/dark cycle and stable temperature (22-23°C) with free access to food and water. All experiments were conducted in accordance with the Sociedade Brasileira de Ciência em Animais de Laboratório (SBCAL) and were approved by the local Ethics in Research Committee (Protocol CEP/UFG 22/2011).

### 2.8. Preparation of Rat Aortic Rings

Rats were anesthetized and subsequently decapitated with a guillotine. The thoracic aorta was removed, cleaned of adhering fat and connective tissue, and then segmented into rings of 4 mm in length. Aortic rings were mounted under 1.5 g resting tension on stainless steel hooks in 9.0 mL warmed (37°C) and oxygenated (95% O_2_ and 5% CO_2_) organ baths containing a modified Krebs-Henseleit solution (pH 7.4; composition in mM: NaCl—130, NaHCO_3_—14.9, KCl—4.7, KH_2_PO_4_—1.18, MgSO_4_·7H_2_O—1.17, CaCl_2_·2H_2_O—1.6, glucose—5.5). The changes in basal tension were recorded by isometric transducers connected to a data acquisition system (World Precision Instruments, Sarasota, FL, USA). The aortic rings were placed under a resting tension of 1.5 g and equilibrated for 60 min. During this period, tension was periodically adjusted to the desired level and the Krebs-Henseleit solution was changed every 15 min.

### 2.9. Measurement of Vascular Effects of the CHE and Its Fractions and Investigation of Mechanisms of Action

Endothelium-intact aorta rings were precontracted with Phe (0.1 *μ*M). When the contractile responses to Phe had reached a plateau (usually after 15 min), cumulative concentrations (0.1 *μ*g/mL to 30 *μ*g/mL) of CHE or organic fractions (HF; CF; EAF or BF) were added to evaluate the vascular effects. Extract and fractions solutions were prepared in 5% DMSO. The vehicle (DMSO) was also tested and the final concentration in all chambers did not exceed 0.1% (v/v). The BF, which showed a vasorelaxant effect similar to CHE, was chosen for the studies of the mechanism of action and hypotensive effect. 

The integrity of the endothelium was tested in each vessel by observing the relaxation response to ACh (10 *μ*M) following precontraction with Phe (0.1 *μ*M). In order to investigate whether BF-induced relaxation was dependent on the integrity of the vascular endothelium, concentration-response curves to BF (0.1 *μ*g/mL to 30 *μ*g/mL) were determined in endothelium-denuded rings precontracted with Phe. The absence of ACh-induced vasorelaxant effects was taken as evidence that the arterial rings were effectively stripped of endothelium. Finally, SNP (1 *μ*M), an NO-donor, was added to produce endothelium-independent relaxation.

In addition, endothelium-intact aorta rings were incubated with L-NAME (100 *μ*M), a nitric oxide (NO) synthase inhibitor, for 30 min. After the incubation period, aorta rings were then stimulated with Phe (0.1 *μ*M) or KCl (120 mM). When the contractile response had reached a plateau, cumulative concentration-effect curves for BF were constructed.

To investigate the role of prostaglandins and K^+^ channels on BF-induced relaxation, concentration-response curves were constructed in the presence of indomethacin (10 *μ*M) (a cyclooxygenase (COX) inhibitor) and TEA (1 mM) (a nonselective K^+^ channel blocker). The involvement of guanylylcyclase on BF-induced relaxation was measured in the presence of ODQ (10 *μ*M), a soluble guanylylcyclase inhibitor. 

The involvement of endothelial receptors in the vascular effects of BF was measured after incubation of atropine (10 *μ*M), a muscarinic receptor antagonist, or pyrilamine (10 *μ*M), a histamine H_1_-receptor antagonist. In each case, vessels were incubated with inhibitors for 30 min prior to being precontracted with Phe. Responses of the tissues to BF were directly compared to those obtained in the same tissues in the absence of the inhibitors (control). 

Endothelium-denuded rings were incubated in Ca^2+^-free high K^+^ (60 mM) depolarizing Krebs-Henseleit solution. A cumulative concentration-response curve to CaCl_2_ (10 *μ*M to 0.1 mM) was established in the presence of either BF or DMSO for 30 min. The results were expressed as percentages of the maximal response for KCl (120 mM) and the curves were statistically compared. In these experiments, no precipitation of calcium was observed at the concentrations used. 

To address any residual or nonreversible effect of BF on contractile or relaxation events, aortic rings (exposed to cumulative concentrations of BF) were washed and allowed for another 60 min interval, before a new exposure to Phe (0.1 *μ*M) and ACh (10 *μ*M).

### 2.10. Evaluation of the Effects of BF on Blood Pressure of Anaesthetized Rats

On the day of experiments, the rats were anesthetized with halothane (2-3% halothane in 100% O_2_) and catheters were inserted into the right femoral vein and artery. After the catheter placement, the rats were removed from the halothane and the anesthesia was maintained with urethane (1.2 g kg^−1^·b.wt., i.v.; Sigma-Aldrich Co., St. Louis, MO, USA). The rats were mounted prone in a stereotaxic apparatus (David Kopf Instruments) with the bite bar set at 3.4 mm below the interaural line. In experiments measuring the aortic blood flow (ABF), miniature ultrasonic transit-time flow probes (Transonic Systems Inc., Ithaca, NY, USA) were placed around the aorta. The body temperature was kept at 37 ± 0.5°C with a thermostatically controlled heated table. 

To register blood pressure, the arterial catheter was connected to a pressure transducer attached to a bridge amplifier. The pulsatile pressure was recorded continuously with an MP150 analog-to-digital converter (Biopac Systems, Inc., Goleta, CA, USA). The mean arterial pressure (MAP) was determined through the pulsate signal with AcqKnowledge software (version 3.7.1.; Biopac Systems). To measure the aortic blood flow (ABF), a flow probe was connected to an ultrasonic transit-time flowmeter (Transonic Systems). Aortic vascular conductance (AVC) was obtained by the ratio between ABF and MAP. Either the extract (i.e., BF) (7.15, 14.3, 28.6, 57.2, 114.4, and 228.8 *μ*g kg^−1^·b.wt., i.v.; in 0.1 mL) or the vehicle (DMSO 5%) was infused through the femoral vein cannula. 

### 2.11. Statistical Analysis

The results are presented as the mean ± SEM of 6–8 experiments. Statistical significance was determined using Student's *t*-test or one-way analysis of variance (ANOVA) followed by Bonferroni's post hoc test when appropriate. A *P* value of <0.05 was considered statistically significant. Analyses were performed using GraphPad Prism version 5.00 for Windows (San Diego, CA, USA).

## 3. Results

### 3.1. Vascular Effects of the CHE and Its Fractions

**Figure 1:**
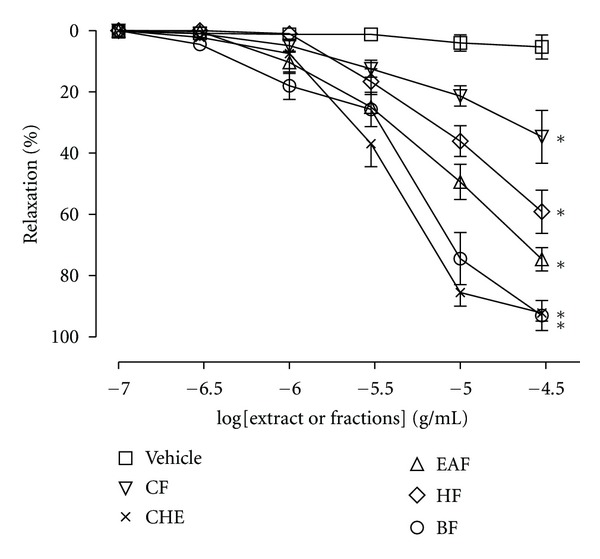
Cumulative concentration-response curves (0.1 to 30 *μ*g/mL) for crude hydroalcoholic extract (CHE), hexane fraction (HF), chloroform fraction (CF), ethyl acetate fraction (EAF), and butanolic fraction (BF) of *C. brasiliense* leaves in rats aortic rings with intact endothelium precontracted with phenylephrine (Phe) (0.1 *μ*M). The results show the mean ± SEM of 6–8 experiments. *P < 0.05 when compared to vehicle (DMSO).

**Figure 2:**
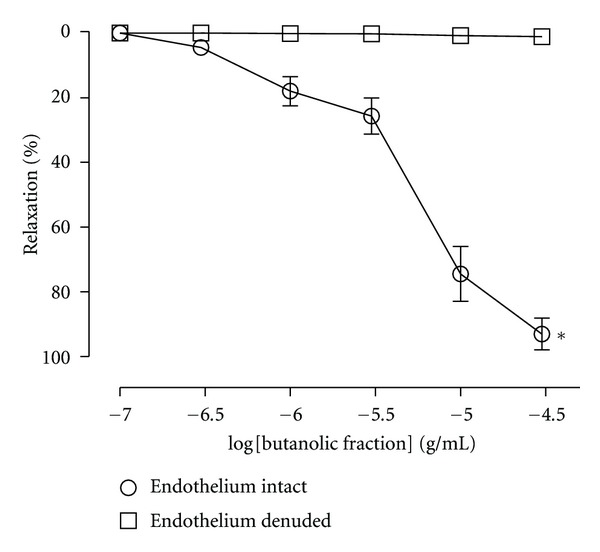
Cumulative concentration-response curves (0.1 to 30 *μ*g/mL) for butanolic fraction (BF) of *C. brasiliense* leaves in rats aortic rings with intact or denuded endothelium precontracted with phenylephrine (Phe) (0.1 *μ*M). The results show the mean ± SEM of 6–8 experiments. *P < 0.05 when compared to endothelium-denuded aortic rings.

**Figure 3:**
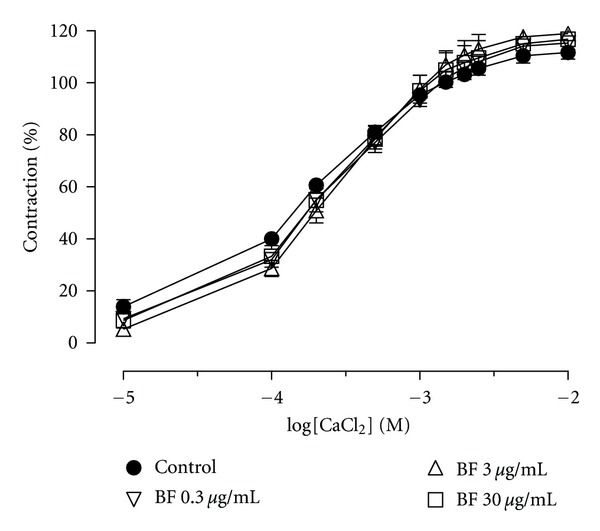
Effect of BF (0.3, 3, and 30 *μ*g/mL) on the contractile responses elicited by incremental addition of Ca^2+^ (10 *μ*M–0.1 mM) to aortic rings depolarized by isotonic KCl (60 mM) in a Ca^2+^-free solution. The concentrations of BF are indicated on the graph. Contractile responses are expressed as the percentage of maximum contraction evoked by KCl (120 mM). The results show the mean ± SEM of 5–7 experiments.

Endothelium-intact aortic rings precontracted with Phe (0.1 *μ*M) relaxed in a concentration-dependent manner in response to the cumulative addition of CHE and organic fractions (HF; CF; EAF or BF). CHE and BF produced potent and similar relaxations (*E*_max⁡_ = 97.4 ± 2.2% and 94.4 ± 2.5%, resp.) (Figure 1). The EC_50_ for CHE and BF was 3.9 *μ*g (2.8–5.4) and 5.6 *μ*g (3.5–8.9), respectively. The EAF, HF and CF elicited a maximum relaxation of 74.7 ± 3.8%, 59.1 ± 7.1% and 34.7 ± 8.6%, respectively. The vehicle had no significant relaxant effect (<5%). Sixty minutes after the extracts were removed from the baths, the effects of either Phe or ACh were similar to those seen before the addition of the extracts (data not shown). Because BF was the organic fraction with the best profile of vasorelaxation, we established cumulative concentration-response curves to this fraction in order to investigate the mechanism of action. Unlike the effect observed in endothelium-intact aortic rings, the BF-induced relaxation was abolished in preparations without endothelial cells (Figure 2). We also found that the endothelium-denuded rings incubating with depolarizing and nominally without Ca^2+^ solution, CaCl_2_ was able to induce contractions that were not inhibited by BF in doses of 0.3, 3, and 30 *μ*g/mL (Figure 3). 

**Figure 4:**
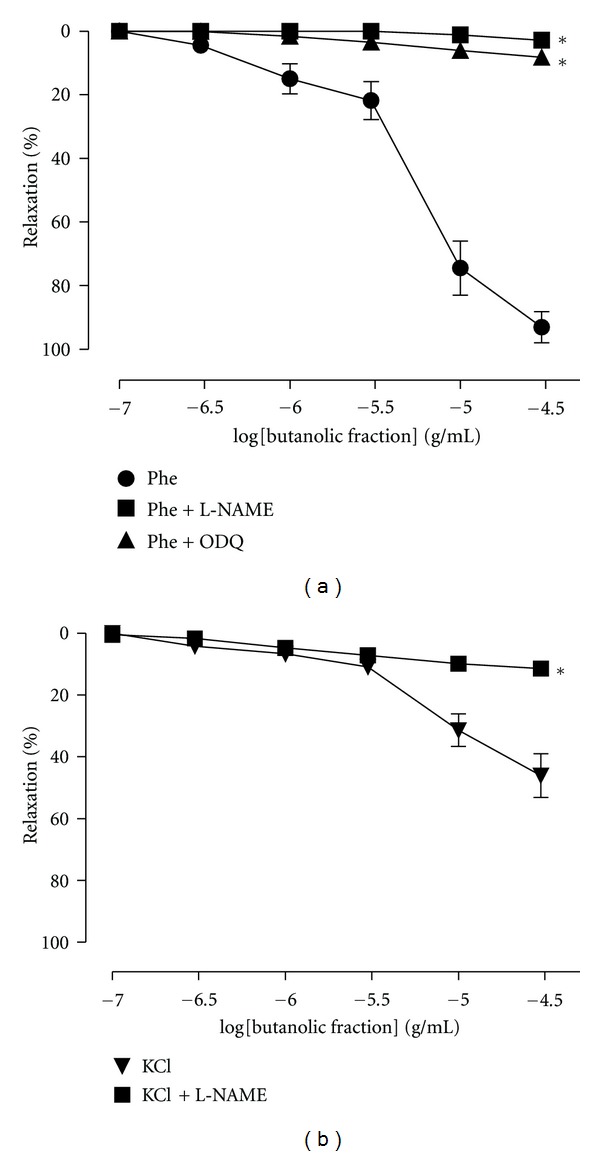
Cumulative concentration-response curves for butanolic fraction (BF) in aortic rings with intact endothelium precontracted with (a) phenylephrine (Phe) (0.1 *μ*M) in the presence of N*ω*-nitro-L-arginine methyl ester (L-NAME) (a nitric oxide synthase inhibitor, 100 *μ*M) or 1H-[1,2, 4]oxadiazolo [4,3-alpha]quinoxalin-1-one (ODQ) (a guanylylcyclase inhibitor, 100 *μ*M) and (b) precontracted with KCl (120 mM) in the presence or absence of L-NAME (100 *μ*M). The results show the mean ± SEM of 6–8 experiments. *P < 0.05 when to control (Phe or KCl).

**Figure 5:**
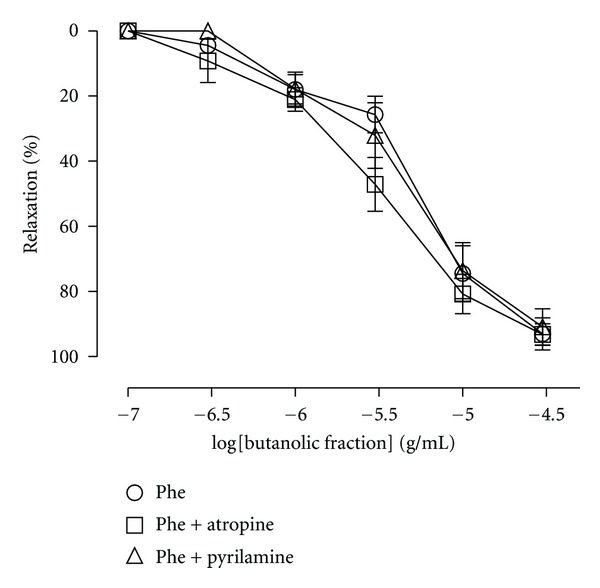
Relaxation evoked by BF before (control curve) and after exposure to pyrilamine (a H_1_ receptor antagonist; 10 *μ*M) or atropine (a muscarinic antagonist receptor; 10 *μ*M) in rat aortic rings. The results are expressed as mean ± SEM of 5–7 experiments.

**Figure 6:**
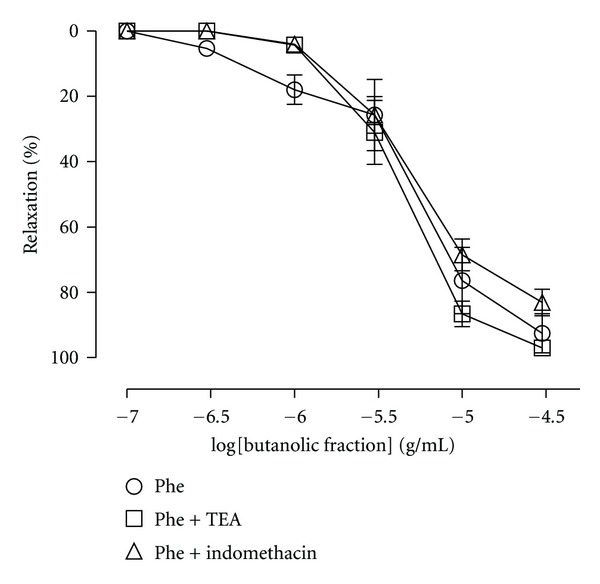
Relaxation evoked by BF in the absence (control curve) or presence of tetraethylammonium (TEA, a nonselective potassium channel blocker; 1 mM) and indomethacin (a cyclooxygenase inhibitor; 10 *μ*M) in rat aortic rings. The results are expressed as mean ± SEM of 6-7 experiments.

In aortic tissue precontracted with Phe (0.1 *μ*M), incubation with L-NAME (NOS inhibitor) or ODQ (a guanylylcyclase inhibitor) was able to completely inhibit the relaxation induced by BF (Figure 4(a)). In intact endothelium precontracted with KCl (120 mM), the BF produced less relaxation (*E*_max⁡_ 63,7 ± 7,0%) compared to that observed with the Phe (*E*_max⁡_ 94,4 ± 2,5%). However, the pretreatment with L-NAME (100 *μ*M) completely inhibited the relaxation produced by BF in aortic rings precontracted with KCl (Figure 4(b)). The effects of atropine and pyrilamine are shown in Figure 5. BF-induced aortic relaxation was not attenuated after incubation with atropine or pyrilamine, respectively. Moreover, the incubation of the arteries with indomethacin (a cyclooxygenase inhibitor) or TEA (a K^+^ channel blocker) did not change the relaxant effect of BF in arteries precontracted with Phe (Figure 6). 

### 3.2. Effects of Intravenous Extract Infusion on Cardiovascular Responses

**Figure 7:**
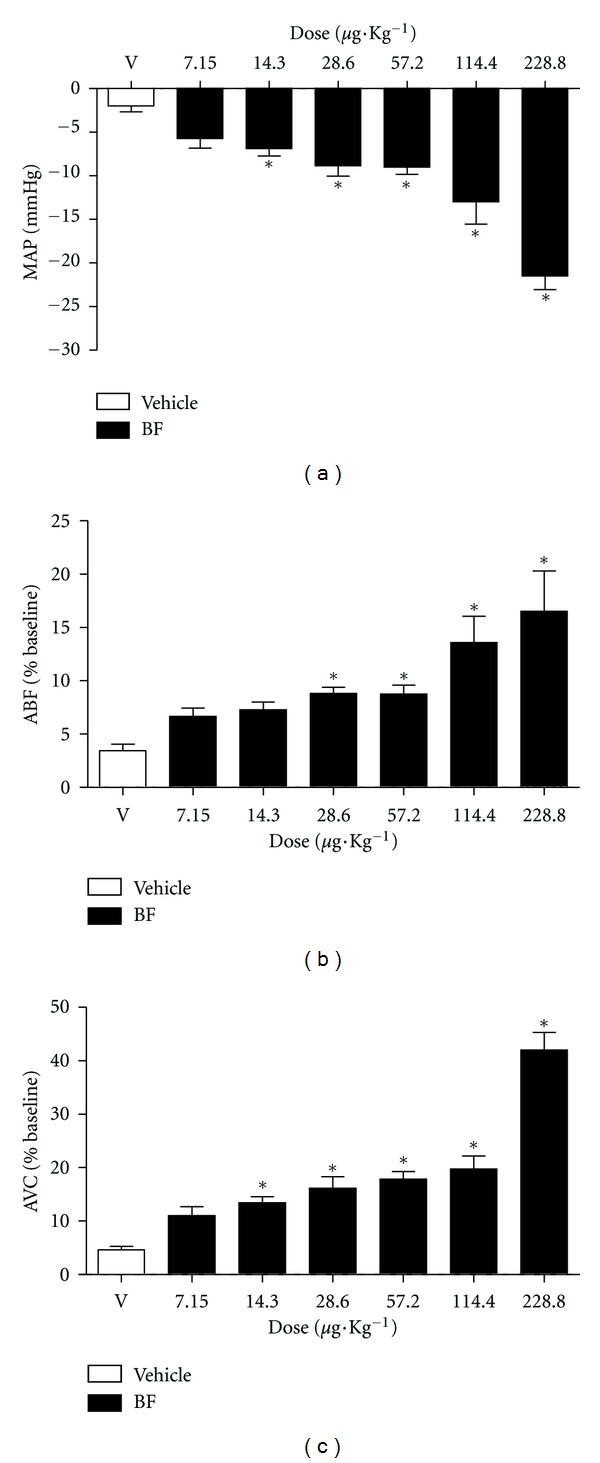
Effects of BF infusion (7.15, 14.3, 28.6, 57.2, 114.4, and 228.8 *μ*g kg^−1^·b.wt., i.v.; in 0.1 mL) on the cardiovascular parameters in anesthetized rats. (a) Mean arterial pressure (MAP). (b) Aortic blood flow (ABF). (c) Aortic vascular conduction (AVC). The results are expressed as mean ± SEM of 6–9 experiments *P < 0.05 when compared to vehicle (DMSO).

In anesthetized rats, infusion of the vehicle did not promote changes in MAP (−2 ± 0.67 mm Hg of baseline values; Figure 7(a)), ABF (3.4 ± 0.6% of baseline values; Figure 7(b)) or AVC (4.6 ± 0.65% of baseline values; Figure 7(c)). Intravenous infusion of the BF (7.15, 14.3, 28.6, 57.2, 114.4, and 228.8 *μ*g kg^−1^·b.wt.) produced a significant dose-dependent hypotension (−5.6 ± 1.1; −6.9 ± 0.9; −8.8 ± 1.2; −9.0 ± 0.9; −13.0 ± 2.6; −21.5 ± 1.6 mm Hg of baseline values, respectively, Figure 7(a)), as well as increases in ABF (6.7 ± 0.8; 7.3 ± 0.7; 8.8 ± 0.5; 8.9 ± 0.8; 13.6 ± 2.5; 16.5 ± 3.8% of baseline values, respectively, Figure 7(b)) and in AVC (11.0 ± 1.7; 13.4 ± 0.1; 16.1 ± 2.2; 17.9 ± 1.4; 19.7 ± 2.5; 42.0 ± 3.3% of baseline values, resp., Figure 7(c)).

### 3.3. Analysis of Butanolic Fraction by LC-MS

**Figure 8:**
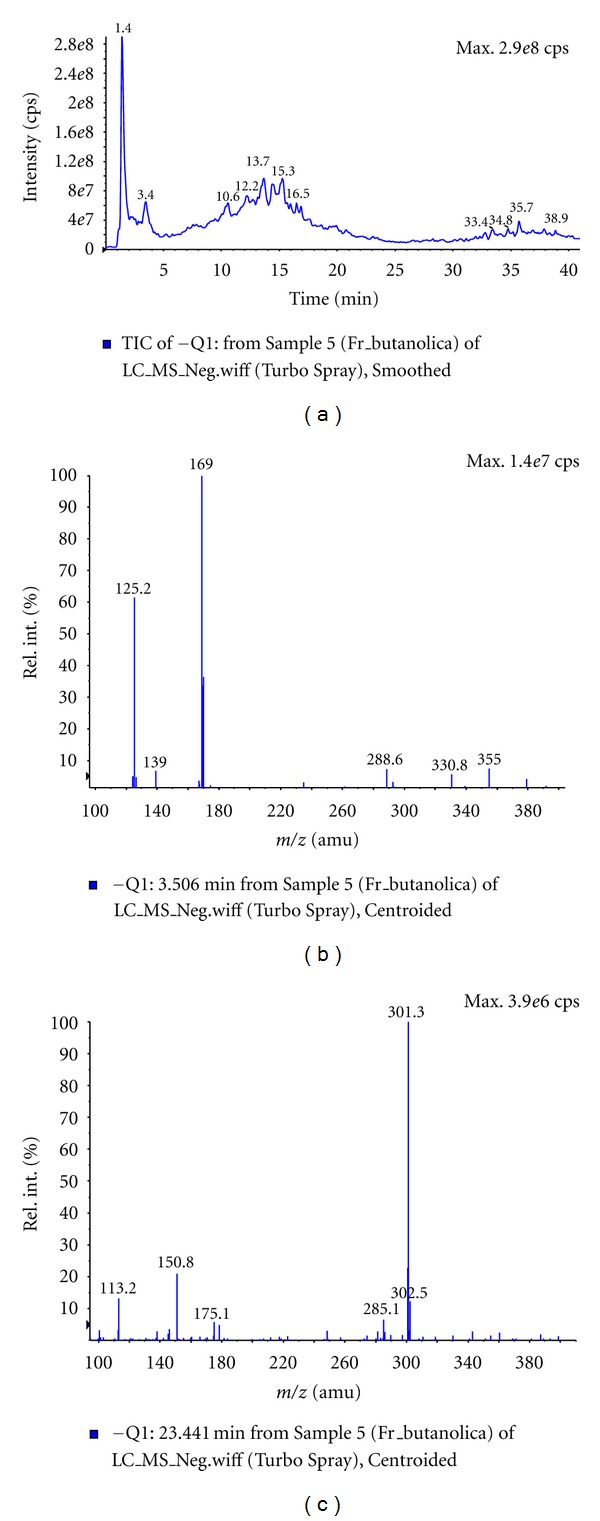
LC-ESIMS analysis of butanolic fraction (BF) from of *C. brasiliense* leaves. (a) Chromatogram of BF in methanol/water solution (70 : 30, v/v) with 1 mM ammonium formate. (b) Mass spectra extracted in *t*_R_ = 3.5 min of chromatogram of BF. (c) Mass spectra extracted in *t*_R_ = 23.4 min of chromatogram of BF.

**Figure 9:**
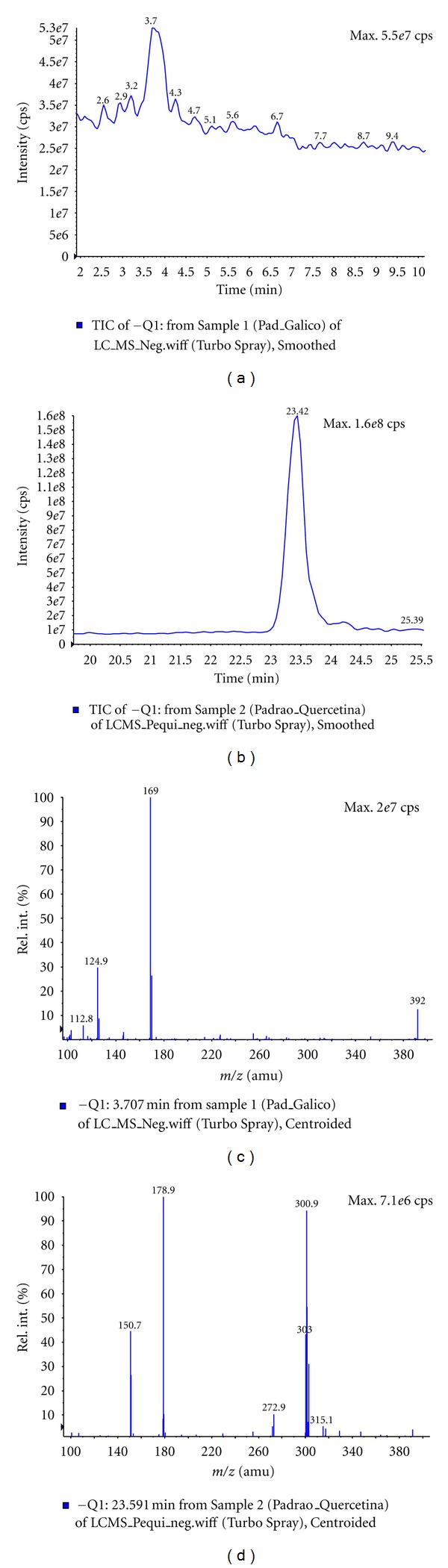
LC-ESIMS analysis of gallic acid and quercetin standards in methanol/water solution (70 : 30, v/v) with 1 mM ammonium formate. (a) Chromatogram of gallic acid standard. (b) Chromatogram of quercetin standard. (c) Mass spectra extracted in *t*_R_ = 23.5 min of chromatogram of gallic acid standard. (d) Mass spectra extracted in *t*_R_ = 3.7 min of chromatogram of quercetin standard.

LC-ESIMS analysis were carried out exploring the representative ions of standards of gallic acid and quercetin in order to confirm the presence or absence of these compounds in butanolic fraction (BF) from *Caryocar brasiliense *leaves. Analysis of BF in the positive and negative mode were obtained to adjust the LC-ESIMS conditions. Posteriorly, standards solutions were injected by LC-ESIMS with the purpose of choosing the most appropriate ionization mode and concentration for the analysis of these compounds in fraction. In the mass spectra extracted from chromatogram in *t*_R_ = 3.5 min and 23.4 min obtained in negative mode experiment of BF was observed the quasimoleculares ions [M − H]^−^ at *m/z* 169.0 and *m/z* 301.3, concordant with observed for gallic acid and quercetin standards, respectively. In addition, there were also observed characteristics fragments ions at *m/z* 125.2 and *m/z* 150.8, obtained by dissociation of quasimoleculares ions [M − H]^−^ of gallic acid and quercetin compounds, respectively, in ESI source (Figures 8 and 9). The quercetin compound was also confirmed in positive mode experiment, which was observed the presence of quasimolecular ion [M + H]^+^ at *m/z* 303.0.

## 4. Discussion

The observation that the butanolic fraction of leaves of *C. brasiliense* is able to fully relax rat aortic rings and dose-dependently reduce the blood pressure of anaesthetized rats is the main finding of this study. In the first stage of this study, we showed that the crude hydroalcoholic extract (CHE) of *C. brasiliense* leaves was able to fully relax rat aortic rings. In the second stage, we found that the BF obtained from CHE showed better vasorelaxation compared to that of other organic fractions (i.e., HF, CF, and EAF) and that it was similar to the effect induced by CHE. Subsequently, the investigation of the mechanism of action and the hypotensive effect was performed with the BF. After the experiments on aortic rings with or without endothelium, we observed that the vasorelaxation effect of BF was dependent on endothelium, since the endothelium-denuded aortic ring preparations were not able to relax after the addition of BF. 

When we constructed a concentration-response curve to CaCl_2_ in a high K^+^ solution before and after incubation with BF, it was not capable of antagonizing the CaCl_2_-induced contractions, suggesting that the BF is not acting as a calcium channel blocker. This result confirms the absence of effect on smooth muscle cells.

The vascular endothelium plays an important role in cardiovascular functions, mainly by controlling arterial tone via synthesis and release of potent vasodilators such as nitric oxide, vasodilator prostaglandins (e.g., prostacyclin) and endothelium-derived hyperpolarizing factor (EDHF) [16]. Accordingly, to verify the involvement of endothelium-derived vasodilators, the effect of L-NAME on the BF-induced vasorelaxation was investigated. We demonstrated that NO is involved in the BE-induced vasorelaxation of aortic rings with intact endothelium because L-NAME (a nitric oxide synthase inhibitor) as well as ODQ (a soluble guanylatecyclase inhibitor) abolished this effect. The finding that BF produced greater relaxation of Phe-contracted aortic rings than KCl-contracted aortic rings (Figure 4) is also consistent with the properties of NO-dependent vasorelaxation [17–19]. It is unlikely that prostacyclin and potassium channels are involved in this effect because indomethacin and TEA (a prostaglandins inhibitor and a potassium channels blocker, resp.) were not able to reduce the vasodilatation effect induced by BF. 

We also investigated the possible mechanism involved in the ability of BF to stimulate NO release. Under physiological conditions, NO is continuously released by endothelial cells as a consequence of the shear stress generated by blood flow. However, its release is also mediated by the activation of muscarinic [17] and histaminic H_1_ [20] receptors, among others. Our results indicate that neither cholinergic nor histaminergic pathways are involved, since the vasorelaxation effect induced by BF was unchanged by incubation with atropine and pyrilamine, respectively.

Consistent with vascular reactivity results, the infusion of BF in anesthetized rats induced an increase of AVC, indicating aortic vasodilation. In addition, this aortic vasodilation may be related, at least partly, to the hypotensive effect induced by BF. 

The exposure of BF did not result in any long-lasting impairment in the contractile or relaxation functions of the aortic rings, and was found to cause a reversible hypotension in rats. This suggests that, at least in the range of concentrations and doses used in this study, this extract does not have deleterious effects. 

In this study, the butanolic fraction of *C. brasiliense* leaves presented a better profile for aortic ring relaxation compared to that of the other organic fractions. Butanol is a solvent of higher polarity and it extracts flavonoids, tannins, and saponins, among others chemical substances [21]. In a previous study, the chemical characterization of the polar components of pequi fruits showed the presence of bioactive components such as gallic acid and quercetin [9]. Similarly, the LC-ESIMS analysis showed the presence of gallic acid and quercetin in BF. The methods applied are simple and direct for rapid identification of phenolic compounds in plant fraction of *C. brasiliense*. It eliminated tedious procedures of separation and purification, providing sufficient information to the confirmation of known compounds when standards mass spectra and fragmentation profile are compared to literature. 

It has been described that quercetin is effective for enhancing the bioavailability of endothelium-derived nitric oxide [22, 23], while gallic acid is known to induce vascular relaxation in higher concentrations [24]. Accordingly, the confirmation of presence of gallic acid and quercetin in BF by LC-MS can help in the understanding of its vasorelaxant effect, since these compounds have been reported in the literature for such activity.

## 5. Conclusions

Our results reveal that *C. brasiliense* possesses both in vivo and in vitro activities and that the vascular effect of BF involves stimulation of the nitric oxide/cyclic GMP pathway. In addition, these findings corroborate previous work performed with volunteer athletes, which demonstrated that the administration of pequi caused hypotension. So, this work contribute to our understanding of the potential use of *C. brasiliense* in pathological conditions, such as hypertension and atherosclerosis.
